# Molecular Uptake of Chitooligosaccharides through Chitoporin from the Marine Bacterium *Vibrio harveyi*

**DOI:** 10.1371/journal.pone.0055126

**Published:** 2013-01-29

**Authors:** Wipa Suginta, Watcharin Chumjan, Kozhinjampara R. Mahendran, Petra Janning, Albert Schulte, Mathias Winterhalter

**Affiliations:** 1 Biochemistry-Electrochemistry Research Unit, Schools of Chemistry and Biochemistry, Institute of Science, Suranaree University of Technology, Nakhon Ratchasima, Thailand; 2 School of Engineering and Science, Jacobs University Bremen, Bremen, Germany; 3 Department of Chemical Biology, Max-Planck Institute of Molecular Physiology, Dortmund, Germany; Swiss Federal Institute of Technology Zurich, Switzerland

## Abstract

**Background:**

Chitin is the most abundant biopolymer in marine ecosystems. However, there is no accumulation of chitin in the ocean-floor sediments, since marine bacteria *Vibrios* are mainly responsible for a rapid turnover of chitin biomaterials. The catabolic pathway of chitin by *Vibrios* is a multi-step process that involves chitin attachment and degradation, followed by chitooligosaccharide uptake across the bacterial membranes, and catabolism of the transport products to fructose-6-phosphate, acetate and NH_3_.

**Principal Findings:**

This study reports the isolation of the gene corresponding to an outer membrane chitoporin from the genome of *Vibrio harveyi*. This porin, expressed in *E. coli*, (so called *Vh*ChiP) was found to be a SDS-resistant, heat-sensitive trimer. Immunoblotting using anti-ChiP polyclonal antibody confirmed the expression of the recombinant ChiP, as well as endogenous expression of the native protein in the *V. harveyi* cells. The specific function of *Vh*ChiP was investigated using planar lipid membrane reconstitution technique. *Vh*ChiP nicely inserted into artificial membranes and formed stable, trimeric channels with average single conductance of 1.8±0.13 nS. Single channel recordings at microsecond-time resolution resolved translocation of chitooligosaccharides, with the greatest rate being observed for chitohexaose. Liposome swelling assays showed no permeation of other oligosaccharides, including maltose, sucrose, maltopentaose, maltohexaose and raffinose, indicating that *Vh*ChiP is a highly-specific channel for chitooligosaccharides.

**Conclusion/Significance:**

We provide the first evidence that chitoporin from *V. harveyi* is a chitooligosaccharide specific channel. The results obtained from this study help to establish the fundamental role of *Vh*ChiP in the chitin catabolic cascade as the molecular gateway that *Vibrios* employ for chitooligosaccharide uptake for energy production.

## Introduction

Chitin, a *β*-1,4-linked homopolymer of *N*-acetylglucosamine (GlcNAc), is the most abundant polysaccharide in marine ecosystems, because it is a major component of the shells of crustaceans and marine zoo-plankton. It has been estimated that multi-million tons of chitin-containing substances are produced annually in the aquatic biosphere [1]. However, there is no substantial accumulation of chitin on the ocean floor. This is because of bioconversion of this mass of biomaterials, primarily by marine bacteria of the family *Vibrionaceae* [2]. These bacteria utilize chitinous materials very efficiently, converting them into organic compounds that then can be used as nitrogen and carbon sources.

The catabolic cascade of chitin utilization by marine *Vibrios* has been demonstrated elegantly in *Vibrio furnissii* [3]–[8] and *V. cholerae* [9], [10]. The cascade incorporates a large number of genes and enzymes, which are orchestrated in a complex signal transduction pathway [9], [11]. Roseman and co-workers previously identified chitoporin (ChiP) from *V. furnissii* [12] and suggested that it acts as a chitooligosaccharide-specific channel, based on their findings that expression of native ChiP was significantly induced when the *V. furnissi* cells were grown in the presence of chitooliogsaccharides (GlcNAc_2–6_). A null mutant of *V. furnissii* ChiP also showed an impaired growth in the culture supplemented with chitotriose. Phylogenic analysis of marine bacteria of the *Vibrionacae* family identified a *chiP* gene in 16 out of 19 species [13]. Such results indicate that this protein is well conserved within this family. DNA microarray expression profiles further confirmed that expression of the *chiP* gene in *V. cholerae* responded positively to chitin oligosaccharides and that the genes responsible for chitin degradation are under the stringent control of the *chiS* regulon [6], [13].

Although ChiP was identified more than a decade ago, its physiological function as a chitooligosaccharide-specific channel remains unproved. Here, we report cloning and recombinant expression of chitoporin (referred to as *Vh*ChiP) from the marine bacterium *V. harveyi* (formerly *V. carchariae*) type strain 650. The physicochemical properties of *Vh*ChiP were determined using a planar black lipid membrane (BLM) reconstitution technique. High-time resolution single channel current recordings, together with liposome swelling assays, provide strong evidence that *Vh*ChiP is a highly specific channel for the molecular uptake of chitin oligosaccharides.

## Methods

### Ethics Statement

The anti-rabbit polyclonal antibody production procedure was approved by the Animal Care Commission of Suranaree University of Technology. Two adult (8-week-old) female rabbits were purchased from the Animal Caring Center, Mahidol University, Bangkok, Thailand. The rabbit was housed in a standard animal facility under conditions of controlled temperature (25°C) and photoperiod (a 12∶12-hour light/dark schedule), with food and water provided ad libitum.

### Bacterial strains and vectors

*V. harveyi* type strain 650 was a marine isolate from Greek sea bass and was a gift from Professor Brian Austin, Heriot-Watt University, Edinburgh, United Kingdom. *E. coli* strain DH5*α* was used for routine cloning and plasmid preparations. pGEM®-T easy vector used for subcloning purpose was a product of Promega (Promega Pte Ltd, Singapore Science Park I, Singapore). The pET23d(+) expression vector and *E. coli* mutant strain BL21(DE3) Omp8 Rosetta were gifts from Professor Dr. Roland Benz, Jacobs University Bremen, Germany. The *E. coli* mutant was genetically engineered to have defective genes encoding the major outer membrane porins: OmpA, OmpC, OmpF and LamB [14] and was therefore suitable for production of recombinant porin.

### Gene identification, cloning and sequencing

A BlastP search using chitoporin from *V. furnissei* (UniProtKB/TrEMBL entry: Q9KK91 and ref. 12) as protein template identified putative chitoporins from several marine bacteria in family *Vibrionaceae*, including a hypothetical protein VIBHAR_01269 (accession number YP_001444474) from *V. harveyi* type strain ATCC BAA-1116 BB120. Therefore, specific oligonucleotides were designed from the hypothetical gene of the BAA-1116 BB120 strain in order to obtain the gene encoding chitoporin from our laboratory strain (*V. harveyi* type strain 650). Genomic DNA was prepared from this bacterium using PureLink™ Genomic DNA Kits (Invitrogen, Gibthai Company Ltd., Bangkok, Thailand) and used as the DNA template for PCR amplification. The oligonucleotides used for amplification were 5′-ATACCATGGCGTCTTACCTAAAGAAAAG-3′ for the forward primer and 5′-AACCTCGAGTTAGAAGTAGTATTCAACAC-3′ for the reverse primer. The PCR product was of the expected size (1.1 kbp) and was cloned into pET23d(+) expression vector using *Nco* I and *Xho* I cloning sites (sequences underlined) following the protocol supplied by the manufacturer. Nucleotide sequences of sense and anti-sense strands of the PCR fragment were determined by automated sequencing (First BASE Laboratories Sdn Bhd, Selangor Darul Ehsan, Malaysia).

### Recombinant expression and protein purification

*E. coli* BL21 (DE3) Omp8 Rosetta host strain was transformed with the plasmid pET23d(+)/*chiP*. Expression and preparation of the recombinant ChiP followed the protocols described by Garavito and Rosenbusch [15] and Rosenbusch [16]. In brief, transformed cells were grown at 37°C in Luria-Bertani (LB) liquid medium containing 100 µg^.^mL^−1^ ampicillin and 25 µg mL^−1^ kanamycin. At an OD_600_ reading of 0.5–0.7, IPTG (isopropyl *β*-D-thiogalactoside) was added to a final concentration of 0.5 mM. Cell growth was continued for a further 6 h and cells were then harvested by centrifugation at 4,500×g at 4°C for 20 min. The cell pellet was resuspended in a buffer containing 20 mM Tris-HCl, pH 8.0, 2.5 mM MgCl_2_, 0.1 mM CaCl_2_, 10 µg^.^mL^−1^ DNase I and 10 µg mL^−1^ RNase A. Cells were lysed on ice by sonication for 10 min (30% duty cycle; amplitude setting 20%) using a Sonopuls Ultrasonic homogenizer with a 6-mm-diameter probe. The recombinant *Vh*ChiP was extracted from the peptidoglycan layer with sodium dodecyl sulphate (SDS) based on the method of Lugtenberg and Alphen [17]. Briefly, SDS was added to the cell suspension to a final concentration of 2% (v/v) and incubation was carried out for 1 h at 60°C with gentle shaking. The crude extract was then centrifuged at 40,000×g for 60 min at 4°C. The pellet, which at this stage included the cell envelopes, was re-suspended in 20 mM phosphate buffer, pH 7.4, containing 0.125% (v/v) octyl-POE (n-octyl polyoxyethylene; ALEXIS Biochemicals, Lausanne, Switzerland), using a Potter-Elvehjem homogenizer. The suspension was incubated at 37°C with gentle shaking for 1 h and then centrifuged at 100 000×g at 4°C for 40 min. The new pellet, now rich in outer membranes, was resuspended in 20 mM phosphate buffer, pH 7.4 containing 5% (v/v) octyl-POE and the suspension incubated at 37°C for 60 min. Insoluble material was removed by centrifugation at 100,000×g at 20°C for 40 min. After exchange of the detergent to 0.2% (v/v) LDAO (lauryldimethylamine oxide; Sigma-Aldrich Pte. Ltd., Singapore) by dialysis, the *Vh*ChiP-rich sample was subjected to ion-exchange chromatography using a Hitrap Q HP prepacked column (5×1 mL) connecting to an ÄKTA Prime plus FPLC system (GE Healthcare Life Sciences, Life Sciences Instruments, ITS (Thailand) Co., Ltd., Bangkok, Thailand). Bound proteins were eluted with a linear gradient of 0–1 M KCl in the phosphate buffer, containing 0.2% (v/v) LDAO. Purity of the eluted proteins was confirmed by SDS-PAGE. Fractions containing only *Vh*ChiP were pooled and the protein concentration was determined using the Pierce BCA protein assay kit (Bio-Active Co., Ltd., Bangkok, Thailand).

### Antibody production and immunological analysis

Production of anti-*Vh*ChiP antiserum was carried out using an in-gel method. Outer membrane fraction extracted by 5% (v/v) octyl-POE was applied to eight wells in parallel on an 8% polyacrylamide gel. Following electrophoresis and Coomassie Blue staining, the proteins were resolved into two bands. The upper band, just above 40 kDa, was identified by mass spectrometry as *E. coli* OmpN, while the lower band, slightly below 40 kDa, was chitoporin (*Vh*ChiP). The lower bands were excised from the gels, combined (ca. 80 µg protein) and homogenized in 200 µL PBS, pH 7.4, then emulsified with 500 µL Freund's complete/incomplete adjuvant (Pierce). The emulsified mixture was injected subcutaneously into a female white rabbit to produce *Vh*ChiP antiserum. Antibody titres and cross-reactivities against other membrane proteins, including *E. coli* OmpF, *E. coli* OmpN and *Burkholderia pseudomallei* Omp38 were checked by Western blotting. Signals representing antibody-protein interaction were detected with HRP-conjugated IgG using the enhanced chemiluminescence method (ECL, Amersham, UK). Anti-OmpN serum was prepared using purified *E. coli* OmpN, its titres and cross-reactivities being tested in the same way as the *Vh*ChiP antiserum.

For expression of native *Vh*ChiP, a 5-mL overnight culture of *V. harveyi* 650 grown in marine medium [18] was transferred to a 2-L flask containing 500 mL of marine medium. The cells were grown at 30°C with agitation until OD_600_ reached 0.6, then 1% (wet w/v) colloidal chitin was added to induce chitoporin expression. Aliquots of 1 mL of cell culture were taken at various time points (1, 2, 3, 4, 5, and 6 h). Cell pellets collected after centrifugation were solubilized in 5× SDS-gel loading buffer, and then analyzed by SDS-PAGE, followed by western blotting.

### Black lipid bilayer measurements and single channel analysis

Black lipid bilayer (BLM) measurements and single channel analysis were performed as described elsewhere [19]–[24]. The lipid bilayer cuvette consisted of two chambers with a 25 µm-thick Teflon film sandwiched in between. The latter had a small aperture of 60–100 µm in diameter across which a virtually solvent free planar lipid bilayer was formed. The chambers were filled with electrolyte solution and an electrode (Ag/AgCl electrodes; World Precision Instruments, Sarasota, FL) immersed on either side of the Teflon film. The electrolyte used was 1 M KCl adjusted to pH 7.5, and buffered by 20 mM HEPES. 1,2-Diphytanoyl-sn-glycero-3-phosphatidylcholine (DPhPC; Avanti Polar Lipids, Alabaster, AL) lipid was used for lipid bilayers formation. In order to form the bilayer first the aperture was pre-painted with 1 µL of 1% (v/v) hexadecane in pentane (Sigma Aldrich). One of the electrodes was used as ground (*cis*) whereas the other electrode was connected (*trans*) to the headstage of an Axopatch 200B amplifier (Axon Instruments, Foster City, CA). Trimeric *Vh*ChiP channel (50–100 ng^.^mL^−1^) was added to the *cis* side of the lipid membrane. At applied transmembrane potentials of ±200 mV, a single channel was frequently inserted within a few minutes. The protein solution in the chamber was gently diluted out by multiple additions of the working electrolyte to prevent multiple insertions. Single channel current measurements were performed with an Axopatch 200B amplifier (Molecular Devices, Sunnywale., CA, U.S.A.) in the voltage clamp mode, with the internal filter set at 10 kHz. Amplitude, probability, and single channel analyses were performed using pClamp v.10.0 software (all from Molecular Devices, Sunnyvale, CA).

To investigate sugar translocation, a chitooligosaccharide was added to either the *cis* or the *trans* side of the chamber to a final concentration of 80 µM. Occlusions of ion flow observed as a result of sugar diffusion through the inserting channel were usually recorded for 2 min. To see the effect of sugar translocation on individual subunit blockages, discrete concentrations of chitohexaose (1, 120, and 400 µM) were tested.

### Liposome swelling assay

Trimeric *Vh*ChiP channel was reconstituted into liposomes as described previously [25], [26]. *E. coli* total lipid extract was used to form liposomes and 15% dextran (MW 40,000) was entrapped in the liposomes. The size of the formed liposomes was checked using a Nano-ZS ZEN3600 zetasizer. The isotonic solute concentration was determined with different concentrations of raffinose solution (prepared in 20 mM HEPES buffer, pH 7.5) added into the protoliposome suspension. The value obtained for isotonic concentration of raffinose was used as an approximation to facilitate the adjustment of isotonic concentrations for different solutes. Twenty microliters of liposome or proteoliposome solution was diluted into 500 µL of the isotonic test solution in a 1-mL cuvette and mixed manually. The initial swelling rate upon addition of the isotonic sugar solutions (maltose, sucrose, maltopentaose, maltohexaose, and chitohexaose) was monitored using a UV-Vis spectrophotometer with the wavelength set at 500 nm. The absorbance change over the first 60 sec was used to estimate the swelling rate (s^−1^) following the equation: Φ = (1/A_i_)dA/dt, in which Φ is the swelling rate, A_i_ the initial absorbance, and dA/dt the rate of absorbance absorbance change during the first 60 s. The swelling rate of each sugar was normalized by setting the rate of arabinose (MW 150.14 Da) to 100%. Values presented are averages obtained from four to six determinations. Protein-free liposomes and proteoliposomes without sugars were used as negative controls.

## Results

### Gene isolation, cloning, sequence analysis and transmembrane topology

The availability of the complete genome sequence of *V. harveyi* type strain ATCC BAA-1116 BB120 in the GenBank® database enabled us to identify an open reading frame that encodes a hypothetical chitoporin (ChiP). To isolate the gene encoding ChiP from the genome of the closely related species *V. harveyi* type strain 650, specific oligonucleotide primers were designed, based on the identified *chiP* gene from the BAA-1116 BB120 strain. The full-length *chiP* cDNA was amplified by the PCR technique. The nucleotide sequence of the identified gene comprises 1,125 bps, which was translated to a putative polypeptide of 375 amino acids, including the 25-aa signal sequence. The theoretical mass of the full-length *Vh*ChiP was 41,089.10 Da, with a predicted *p*I of 4.09. BLAST searching of the translated *Vh*ChiP sequence gave high-score hits with putative chitoporin of several species in the family *Vibrionaceae* in the SwissProt/UniProtKB database.

**Figure 1:**
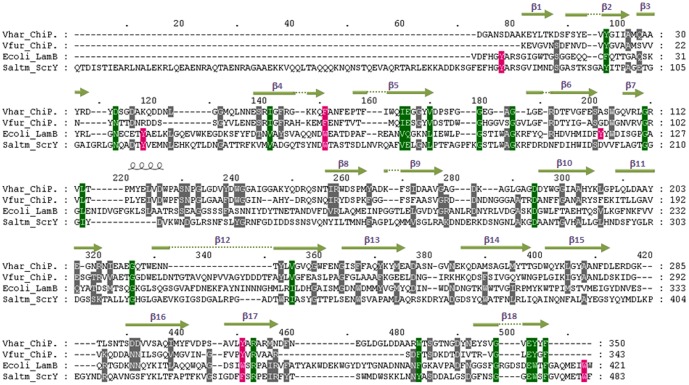
Alignment of the putative *V. harveyi* chitoporin sequence with other sugar-specific porins. Amino acid sequences of *V. furnissii* chitoporin (Q9KK91), *E. coli* LamB or maltoporin (P02943), and *S. typhimurium* ScrY (P22340) were retrieved from the SwissProt/UniProtKB protein databases, aligned using “CLASTALW” algorithm in the DNASTAR package, and displayed in Genedoc. The secondary structure of *Vh*ChiP was constructed by ESPript v. 2.2 according to the structure of *Delftia acidovorans* Omp32 (pdb 2GFR and ref 37). The residues that are aligned with Y6, Y41, Y118, W74, W358, and W420 of *E. coli* LamB are shaded in magenta. Green shading refers to amino residues conserved within the four sequences. β-strands are represented as green lines with an arrow.

*Vh*ChiP shows low sequence identity (<20%) with other functionally characterized outer membrane porins, such as *E. coli* OmpF (P02931), *E. coli* OmpC (P06996), *E. coli* OmpA (P0A910), *E. coli* OmpN (P47747), *Pseudomonas fluorescens* OprD (Q3LAG8), and *Neisseria gonorrhoeae* PorB (Q5XKX0). Fig. 1A presents amino acid sequence alignment of *Vh*ChiP with chitoporin from *V. furnissii* (accession number 09KK91) [12], *E. coli* LamB or maltoporin (maltose-specifc porin) (P02943) [27], and *Salmonella typhimurium* ScrY (sucrose-specific porin) (P22340 [28]. The sequence identity of *Vh*ChiP with *V. furnissii* chitoporin is 40%, while it shows remarkably low identity with other sugar-specific porin: LamB (15.3%), and ScrY (12.9%). It is also only 15.7% identical to a carbohydrate-selective porin *Pseudomonas aeruginosa* OprB [29], [30]. In LamB, six aromatic residues (Y6, Y41, W74, F229, W358 and W420) located in the pore lumen form a polar track, which aids ion and sugar transport [31]–[34]. Y118, on the other hand, controls the central constriction of the LamB channel [35], [36].

Sequence alignment (Fig. 1) shows that the residues Y6, Y41, W74, W358 and W420 of LamB are well aligned with Y78, Y118, W151, F435 and W482, respectively, of ScrY. In marked contrast, *Vh*ChiP displays substantial sequence dissimilarities with both LamB and ScrY. Only two residues in LamB (W74 and W358) are aligned with F64 and Y310 of *Vh*ChiP. Furthermore, Y118 of LamB shows no match with any aromatic residue of *Vh*ChiP, which indicates that the functionality of pore constriction by Y118, as found in LamB, is governed by a different residue located elsewhere in the *Vh*ChiP sequence.

**Figure 2:**
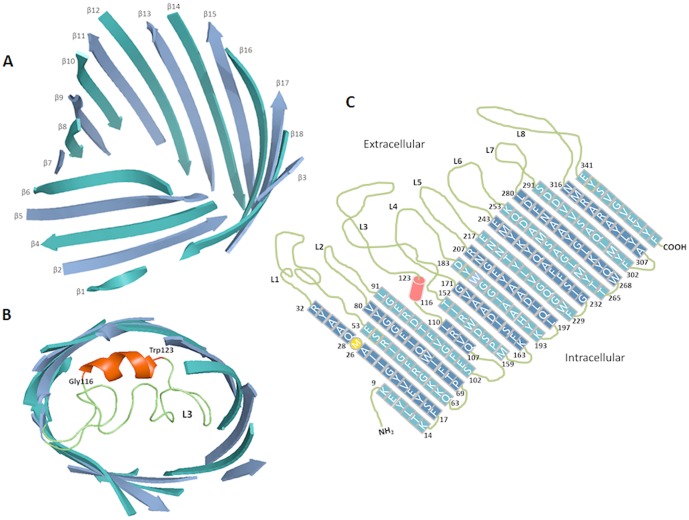
The Swiss-Model 3D-structure of *V. harveyi* chitoporin. A) Side view of a ribbon representation of *Vh*ChiP. The homology structure was constructed by the SWISS-MODEL program using an automated mode (http://swissmodel.expasy.org/). The x-ray structure of *D. acidovorans* Omp32 (pdb 2GFR) was selected as structure template (see texts). B) Top view of the modeled structure, showing L3 as the pore-confining loop with a short helix consisting 8 amino acids (G116-W123) presented in red. C) Transmembrane domains of *Vh*ChiA were depicted based on the homology structure (Fig. 2A–B) and the structure-based alignment (Fig. 1).

Submission of the putative sequence of *Vh*ChiP through the Swiss-Model database generated a structural model of *Vh*ChiP (Fig. 2) using *Delftia acidovorans* Omp32 as template (pdb 2GFR) [37]. Compared with all porins with known 3D-structures, *Vh*ChiP is closest to Omp32 with sequence identity of 20.5%. Fig. 1 shows the secondary structural features of *Vh*ChiP, which are similar to those of most Gram negative bacterial porins, with 18 β-strands forming a barrel structure (Fig. 2A). These predicted 18 anti-parallel β-strands make up only 16 putative membrane-spanning domains, as strand β2 is connected with β3 and forms the first transmembrane domain, whereas strand β1 with β18 are part of the last domain (Figs. 1 and 2A). The predicted transmembrane topology (Fig. 2C) indicates considerable irregularity of the extracellular loops (L1–L8), while the eight periplasmic turns are short and of similar length. The longest extracellular loop (L3), comprising 41 amino acids (G111→N151), lies between strands β7 and β8. A typical right-handed α-helix is found at the early part of L3 at positions P116 to W123 (Fig. 2B). This loop, known as a pore-confined loop, is responsible for the size-selectivity of sugar-specific porins (LamB and ScrY) [28], [31] and general diffusion porins [38], [39].

### Recombinant expression, purification and mass identification

**Figure 3:**
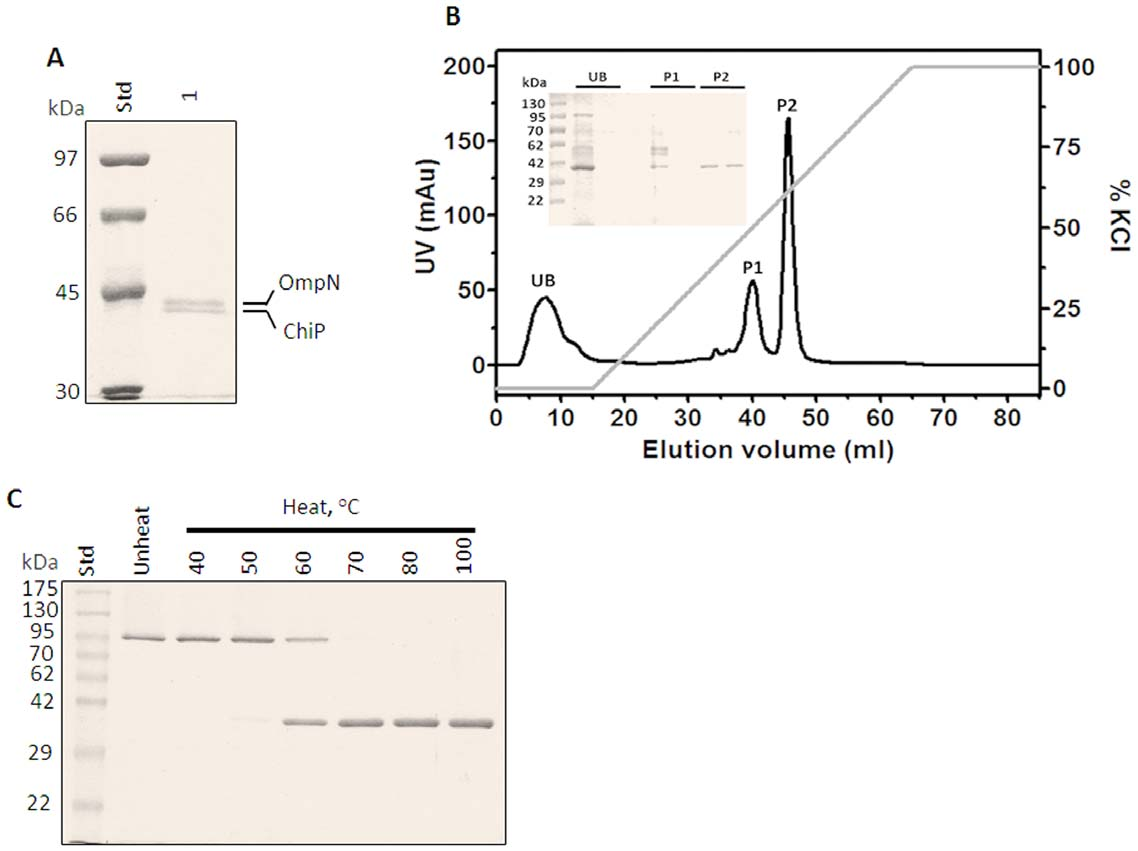
SDS-PAGE analysis of *V. harveyi* chitoporin. A) SDS-PAGE of outer membrane proteins extracted with 2% (w/v) SDS, followed by 5% (v/v) octyl-POE. *E. coli* OmpN and *Vh*ChiP bands were identified by mass spectrometry. B) Chromatographic profile of *Vh*ChiP purification with a Hitrap Q HP prepacked column (5×1 mL) connecting to an ÄKTA Prime plus FPLC system. The column was eluted with a linear gradient of 0–1 M KCl. SDS-PAGE analysis of unbound (UB) and bound fractions P1 and P2 is shown in an inset. C) Heat stability of *Vh*ChiP. The purified ChiP was subjected to different temperatures (40–100°C) and then run on a 10% polyacrylamide gel.

After the correct nucleotide sequence was confirmed, the full-length *chiP* DNA obtained from PCR amplification was cloned into pET23D(+) expression vector, which was ready to be expressed in *E. coli* BL21(DE3) Omp8 Rosetta strain. The recombinant protein was expressed with the 25-amino acid *N*-terminal signal sequence attached, to aid protein targeting to the bacterial cell envelope. After proteolytic removal of the signal sequence, the mature *Vh*ChiP contains 350 amino acid residues and has a predicted MW of 38,508.97 Da. After cell-wall extraction by SDS, following 0.125% (v/v), and then 5% (v/v) octyl-POE, the solubilized fraction contained enriched *Vh*ChiP and a contaminant, which was later identified as *E. coli* OmpN. SDS-PAGE analysis (Fig. 3A) revealed two major protein bands. The upper band migrated close to 40 kDa and the lower band migrated to slightly lower than 40 kDa. Identification of tryptic peptides by high resolution ESI MS gave a primary hit with gi | 3273514 porin OmpN from *E. coli* for the higher MW band, while the lower protein band was identified as gi|28897534 putative chitoporin from *V. parahaemolyticus* RIMD 2210633, as well as gi|153834464 outer membrane protein from *V. harveyi* HY01, and gi|156973567 hypothetical protein from *V. harveyi* ATCC BAA-1116. Given that no functionally-identified chitoporin of the *V. harveyi* species is available in the NCBInr database, we assume that the identified peptides of the lower MW protein were derived from chitoporin (see Fig. S1: nine tryptic peptides are unambiguously identified within the internal segments of the putative *Vh*ChiP sequence).

After several attempts to remove OmpN contamination, we discovered that OmpN was solubilized in 5% octyl-POE, but not in 3%. Therefore, later batches of *Vh*ChiP were prepared in 3% octyl-POE so that OmpN remained in the precipitate. To obtain highly purified *Vh*ChiP for functional characterization, the detergent-extracted *Vh*ChiP was further purified by ion exchange chromatography using a HiTrap DEAE FF column. Fig. 3B shows a chromatographic profile from *Vh*ChiP purification. After removal of the unbound fraction (‘UB’), the bound proteins were then eluted in two peaks (‘P1’ and ‘P2’) when a linear gradient of 0–1 M KCl was applied. SDS-PAGE analysis shows that *Vh*ChiP was in the second peak (P2) and the protein was purified to homogeneity (Fig. 3B, inset) by ion-exchange chromatography. The pooled sample from peak P2 was heat-treated at different temperatures for 10 min, and then analyzed by SDS-PAGE. Fig. 3C shows migration of the purified *Vh*ChiP to above 95 kDa, corresponding to the trimeric form, when unheated (lane 1). The trimer remained intact when the temperature was raised to 40°C, but began to dissociate at 50°C. At 60°C, more than half of the *Vh*ChiP trimers were dissociated to monomers and at 70°C or above, no trimers remained. These results indicate that *Vh*ChiP is a heat-sensitive, SDS-stable trimer; each subunit has apparent MW of approximately 39 kDa, consistent with the predicted MW of the translated polypeptide lacking the signal sequence.

### Immunoblotting and endogenous expression of VhChiP

**Figure 4:**
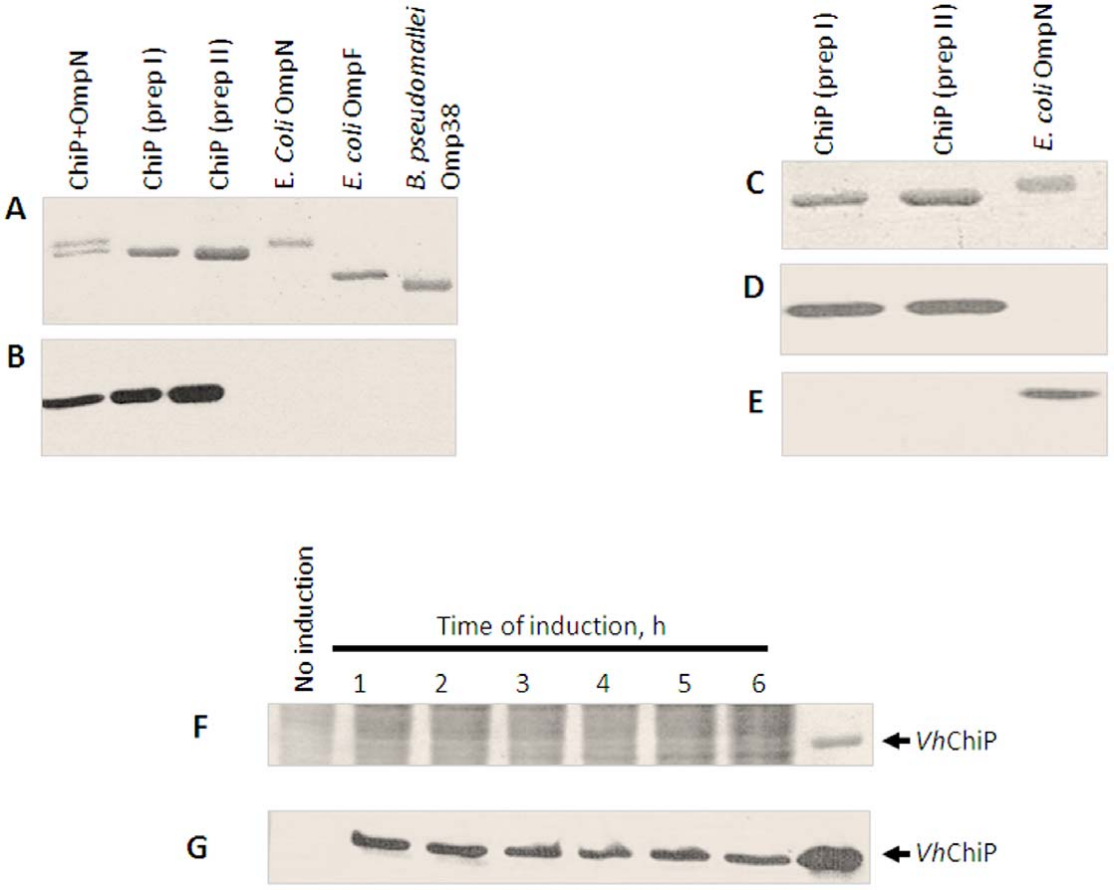
Immunoblot analysis of *V. harveyi* chitoporin. A–B: Cross-reactivity of *VhChiP* antiserum with other outer membrane porins. A. Coomassie blue-stained SDS-polyacrylamide gel, and B. The corresponding immunoblot detected with anti *Vh*ChiP antibody C–D: Cross-reactivity of *Vh*ChiP and *E. coli* OmpN with anti *Vh*ChiP and anti OmpN antibodies. C. Coomassie blue-stained SDS-polyacrylamide gel, D. and E. The corresponding immunoblots showing cross-reactivity with anti *Vh*ChiP antiserum, and anti OmpN antiserum, respectively. F–G: Endogenous expression of chitoporin in *V. harveyi* F. Coomassie blue-stained SDS-polyacrylamide gel, and G. Immunoblot of cell lysate of *V. harveyi* cultured in the presence of 1% (w/v) colloidal chitin at various times of 1–6 h.

To ensure that the recombinant protein obtained was chitoporin and not contaminating OmpN, which was co-expressed by the *E. coli* host strain Omp8 Rosetta, polyclonal antibodies against OmpN and *Vh*ChiP were raised independently. Fig. 4A shows a Coomassie Blue stained gel of different porins, corresponding to the immunoblot with anti-*Vh*ChiP antiserum (Fig. 4B). It is clear that the antibody recognized only the *Vh*ChiP band (Fig. 4A lower band and lanes 2 and 3), but not *E. coli* OmpN (lane 1, upper band and lane 4), *E. coli* OmpF (lane 5) and *B. peudomallei* Omp38 (lane 6). The results suggest no cross reactivity of anti-*Vh*ChiP antibody with other porins. Fig. 4C–E further confirmed that there was no cross-reactivity of the anti-*Vh*ChiP serum with OmpN and anti-*E. coli* OmpN serum with *Vh*ChiP. Anti-*Vh*ChiP serum recognized only *Vh*ChiP (Fig. 4D, lanes 1 and 2), and correspondingly, anti-OmpN serum reacted only with OmpN (Fig. 4E, lane 3).

To determine whether expression of native chitoporin in *V. harveyi* type strain 650 was controlled by the chitin-induced operon, expression profiles of *Vh*ChiP were evaluated after the bacterial cells were grown in the presence of chitin. Fig. 4F shows a Coomassie stained gel of the cell lysates prepared at different times of induction, while Fig. 4G shows the corresponding immunoblot with anti-*Vh*ChiP antibody. It is seen that the antibody reacted with the protein bands in the position of purified *Vh*ChiP when the cells were exposed to 1% (w/v) colloidal chitin for 1 h or more. No positive signal was detected in the lysate prepared from the cells grown in the absence of chitin. We also observed chitoporin expression in the *V. harveyi* cells after induction with crystalline α-chitin, but the signals were not as strong as when colloidal chitin was used (data not shown).

### Single channel properties of VhChiP and chitin oligosaccharide translocation

**Figure 5:**
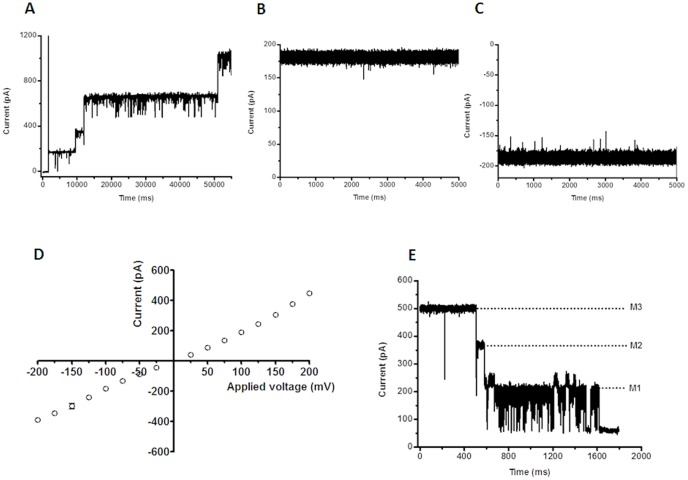
Single channel recordings of chitoporin in artificial lipid membranes. Trimeric *Vh*ChiP was expressed in *E. coli* BL21 (DE3) Omp8 Rosetta mutant, lacking major intrinsic porins. The protein was isolated by SDS-extraction, and then solubilized with 3% (v/v) octyl-POE. The protein was further purified by ion exchange chromatography as described in the text. The BLM measurements were carried out in the electrolyte containing 1 M KCl in 20 mM HEPES, pH 7.5. The protein was added on the *cis* side of the chamber. A. Multiple insertions of *Vh*ChiP were induced at an applied potential of +100 mV. B. Typical ion current trace of a single channel at fully-open state of *Vh*ChiP at applied voltage of +100 mV; and C. at −100 mV. The ion currents were normally recorded for a period of 120 s. D. Analysis of current-voltage (I–V) relationship. The average current values were obtained by stepwise ramping of the potential, preformed in triplicate. E. Three-step closure, induced by increasing the applied voltage to +200 mV.

The pore-forming properties of *Vh*ChiP were investigated at the molecular level using a planar lipid bilayer (BLM) set-up for ion current recordings. The signals for functional analysis were acquired on application of a small potential across two Ag/AgCl wires, one either side of an artificial bilayer of diphytanoylphosphatidylcholine (DPhPC) in 1 M KCl (pH 7.5), and the parallel measurement of the electrostatically driven ion (current) flow through the normally non-conducting lipid membrane, on the inclusion of single pore-forming units. Reconstitution of trimeric *Vh*ChiP into a previously formed lipid bilayer membrane was reproducibly obtained through the addition of a small amount of the purified protein to the bulk phase of the membrane-bathing KCl solution on one or other side of the bilayer. Membrane insertions of *Vh*ChiP were visible in the continuous current recordings as well-defined, step-like increases of about +/−180 pA per protein entity at +/−100 mV transmembrane potential (Fig. 5A). At higher concentrations of *Vh*ChiP (µg^.^mL^−1^) added in the measuring buffer, multiple insertions of *Vh*ChiP were frequently seen and the resultant current traces displayed numerous fluctuations due to transient channel closures. However, the addition of much lower concentrations of added protein (<1 ng^.^mL^−1^) resulted in the incorporation of a single protein molecule in more constantly open state and this was the favored situation for inspecting the *Vh*ChiP single channel conductance and chitin oligosaccharide translocation. Figure 5B and 5C are characteristic examples of membrane current recordings (5 s out of 120 s measuring time) from individual *Vh*ChiP trimers inserted in a DPhPC bilayer in 1 M KCl under applied transmembrane potentials of +100 and −100 mV, respectively. The traces indicate that the inserted *Vh*ChiP channel is fully open, with a stable ionic current over the time of recording. Occasionally transient current deflections occur as one of the three subunits apparently closes and opens rapidly in a stochastic manner. In multiple measurements, single reconstituted trimeric *Vh*ChiP channels showed an average conductance of 1.8±0.13 nS (n = 50) in 1 M KCl (pH 7.5). As with many other bacterial porins, currents through DPhPC-incorporated *Vh*ChiP pores followed Ohm's Law, being directly proportional to the applied voltage over the range ±200 mV (Fig. 5D). Finally, *Vh*ChiP channels showed the typical voltage gating properties of bacterial porins and closed in a characteristic three-step fashion upon abrupt application of higher voltages (Fig. 5E). The threshold potential (critical voltage) inducing the trimeric closure of the channels was found to be ±150 mV, while at less than or equal to 100 mV the channels were not affected by gating perturbations and so were suitable for studies on chitin oligosaccharide translocation.

**Figure 6:**
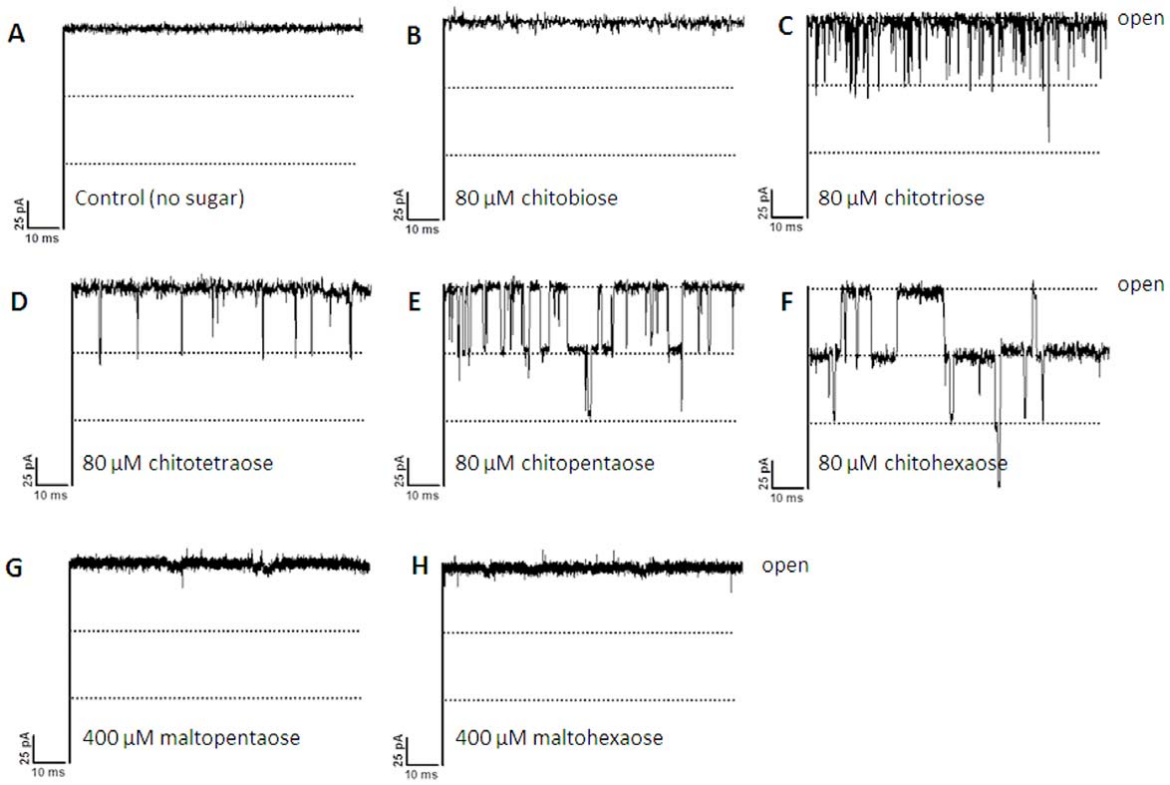
Effect of chitooligosaccharides on chitoporin ion currents. A single channel of *Vh*ChiP was inserted in the artificial membrane in A. a fully open state. Then, chitooligosaccharide: chitobiose, -triose, -tetraose, -pentaose, and -hexaose were added on the *cis* side of the chamber to a final concentration of 80 µM. Control recordings were made with maltopentaose and maltohexaose at a concentration of 400 µM. Ion current fluctuations were monitored for 120 s at applied voltages of ±100 mV. Here, only ion traces for +100 mV are presented.

Chitoporin has been proposed to facilitate the movement of chitin degradation products from the extracellular into the periplasmic space of marine *Vibrios* [12], [13] before they are further transported to the cytoplasm and used as an energy source. To test this function we performed experiments to investigate the effects of chitooligosaccharides of various sizes (see Fig. S2 for the chemical structure of chitobi-, tri-, tetra-, penta-, and hexaose) on fully-open pores of *Vh*ChiP in artificial phospholipid bilayer membranes. Fig. 6 shows current recordings from single *Vh*ChiP channels with all the tested chitooligosaccharides (Fig. 6A–F) as well as those acquired in comparative trials with the structurally related maltopentaose and maltohexaose (Fig. 6G and H). With no chitosugars in the measuring buffer (Fig. 6A), the ion current through a fully open *Vh*ChiP trimer was stable and the standard value of ∼180 pA was measured with a transmembrane potential of +100 mV. The response of the system to the addition of the set of chitosugars was diverse. For instance, no transient decreases were observed when the reconstituted *Vh*ChiP was exposed to chitobiose (Fig. 6B). The current traces obtained had, however, slightly greater noise levels than controls without added solute. In marked contrast, the presence of higher MW chitosugars (GlcNAc_4,5,6_) in the solution on the *cis* side of the membrane produced clear short-lived downward current deflections (Fig. 6C to F). These correspond to the time-resolved blockade of the trimeric pores of *Vh*ChiP by individual chitooligosaccharide molecules that physically obstruct the channels in course of contact. Occlusion of ion flow during sugar diffusion apparently occurred as a reversible process by which each of the brief current decreases was caused by a single sugar molecule entering the *Vh*ChiP channel and leaving it very shortly later. Characteristic current traces for 80 µM chitotriose and chitotetraose showed that no more than one of the three subunits of a *Vh*ChiP trimer was blocked by such chitosugars, the other two remaining unaffected during that period (Fig. 6C and D). The frequency of the single subunit blockades was considerably higher for the triose than for the tetraose. At the same concentration, diffusion of chitopentaose also caused two-subunit blockage (Fig. 6E) and with chitohexaose, even blockage of all three channel subunits could be observed (Fig. 6F). Chitooligosaccharides were also added into the solution on the *trans* side of the bilayer membrane. As with sugar supplementation on the *cis* side, distinct channel blockades were observed in the corresponding membrane current recordings; however, for the same solute concentration the blocking effect was slightly less pronounced. The magnitude of the sugar-induced current depressions is the same for all compounds, corresponding to the quantized blockade of individual subunits; however, the shorter the oligosaccharide, the shorter the time of current blockage. Importantly, the exposure of single *Vh*ChiP channels to maltopentaose and maltohexaose did not cause the transient drops of ion flow that were observed with the chitosugars, even when five times higher concentrations (400 µM) of the maltosugars were used (Fig. 6G and H, respectively).

**Figure 7:**
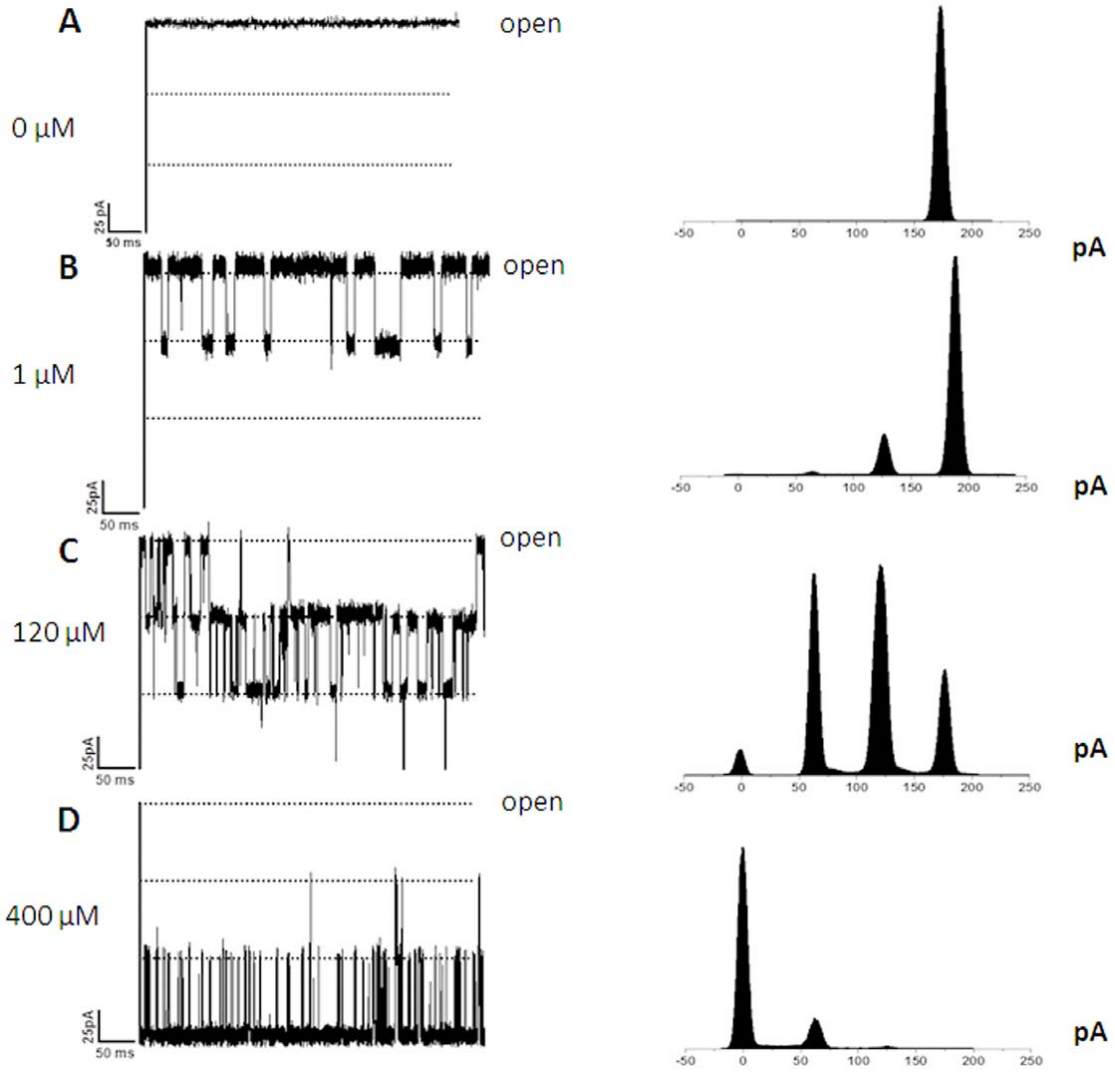
Effects of chitohexaose diffusion on subunit closure. The fully open *Vh*ChiP channel was exposed to different concentrations of chitohexaose (A–D). Right panel: the original traces displaying ion current blockade. Left panel: the corresponding frequency/current histograms, reflecting discrete changes in the subunit conductance upon sugar diffusion through the channel.

BLM trials with different chitosugars identified chitohexaose to be most potent in terms of pore obstruction (Fig. 6). Chitohexaose was thus chosen for evaluating the concentration dependence of chitosugar-induced *Vh*ChiP blockade. Membrane current recordings were taken for the same single channel, while the chitohexamer concentration was progressively increased from 0 µM to 1, 120 and 400 µM, respectively. Fig. 7 shows the original membrane current measurements (A–D, left panel) together with a statistical analysis of the raw data as current magnitude histograms (A–D, right panel). Clearly, the open probability of the channel decreases with increased concentrations of the sugar. On addition of 1.0 µM chitohexaose to the *cis* side of the chamber, the protein channel instantaneously transformed from being constantly fully open (Fig. 7A) to a state in which one subunit of *Vh*ChiP was temporarily occluded (Fig. 7B). This is shown by a decrease of the channel conduction by one-third of the full conductance. As its concentration was raised to 120 µM (Fig. 7C), the sugar began to occupy two subunits, decreasing the conductance by two-thirds. At this concentration, occupation of the third subunit was periodically observed, with the channel conductance reduced to zero. At 400 µM chitohexaose (Fig. 7D), two of the three subunits were constantly blocked, and the effect of increased chitohexaose concentration on the third subunit was apparent. The probability of complete closure of the trimeric channel was approx. 0.8, indicating that the *Vh*ChiP channel was nearly saturated by chitohexaose at this concentration.

The most likely explanation for the short-term inhibition by chitooligosaccharides of ion conduction by *Vh*ChiP is that these molecules permeate the membrane through *Vh*ChiP. Bulk entry of chitooligosaccharides into proteoliposomes containing *Vh*ChiP was therefore investigated by liposome swelling measurements.

### Assay of sugar permeation by liposome swelling

**Figure 8:**
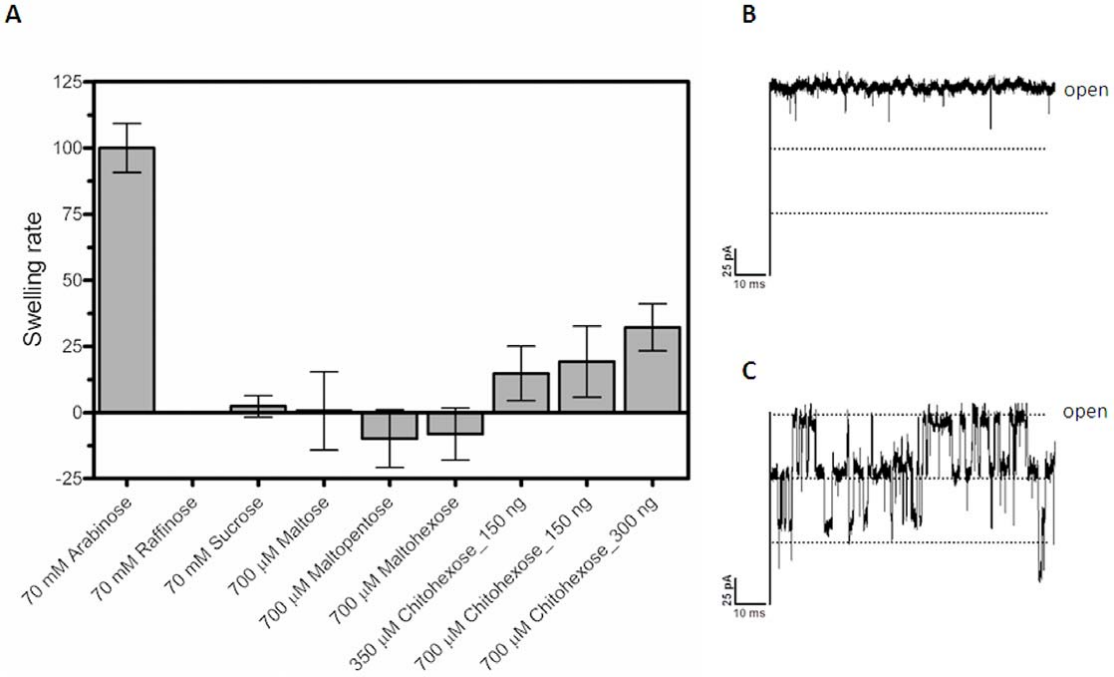
Liposome swelling assays. Multilamellar liposomes, prepared as described in the text, were reconstituted with purified *Vh*ChiP (150 or 300 ng). The isotonic concentration was defined as the concentration of raffinose added into the proteoliposome suspension that did not cause change in absorbance at 500 nm for a period of 60 s. Permeation of different types of sugars through *Vh*ChiP reconstituted liposomes were then tested. A) The swelling rates were normalized, with the rate of swelling in arabinose set to100%. Values presented are averages of 4–6 independent experiments. B) BLM measurement of *Vh*ChiP ion current with the isotonic concentration of raffinose (70 mM) added. C) BLM measurement of *Vh*ChiP in the presence of 70 mM raffinose and 200 µM chitohexaose.

High resolution ion conductance measurements were complemented by proeoliposome swelling assays, which determined the permeation of sugar molecules through *Vh*ChiP channels reconstituted into liposomes. Diffusion rates of sugars through *Vh*ChiP channels determined by these assays indicate influx of solutes into the proteoliposomes. The liposome swelling can be visualized by recording changes in the scattering signal of the liposome solution, using a spectrophotometer. Under isotonic conditions, the scattering signal remains constant throughout the measuring time, indicating neither swelling nor shrinking of the proteoliposomes. In the case of a solute permeation into the proteoliposomes, the total solute concentration inside the vesicles rises, driving the influx of water through the channels and swelling is detected as a decrease in absorbance. It is important to note that the rates of swell provide relative numbers to assess how the translocation varies from one sugar to the other. Here, we used raffinose, a branched sugar (MW 504.42) that is unable to diffuse through the porins and arabinose, a small sugar (MW 150.13) that always permeates through the channel, for comparison. Fig. 8A is an illustration of the swelling of liposomes exposed to chitohexaose, which was the sugar found to be the most potent channel blocker in membrane current measurements. The swelling rates in raffinose, sucrose, maltose, maltopentaose and maltohexaose are included for comparison. When normalized to the swelling rate of arabinose (set to 100%), only chitohexaose at low concentrations (350 and 700 µM) was found to permeate through *Vh*ChiP, and increases of internal osmolality occurred in a concentration-dependent manner. Fig. 8B is *Vh*ChiP single-channel current measurements in the presence of raffinose. The raffinose alone did not cause channel blockage at up to 70 mM, while further addition of a much lower concentration of the chitohexaose (200 µM) to the same bilayer, after the negative results with the raffinose were obtained, immediately produced current deflections (Fig. 8C). Observable swelling of the proteoliposomes apparently reflects permeation of chitohexaose through the embedding *Vh*ChiP pores in the lipid vesicles, but was not significant with other sugars.

## Discussion

**Figure 9:**
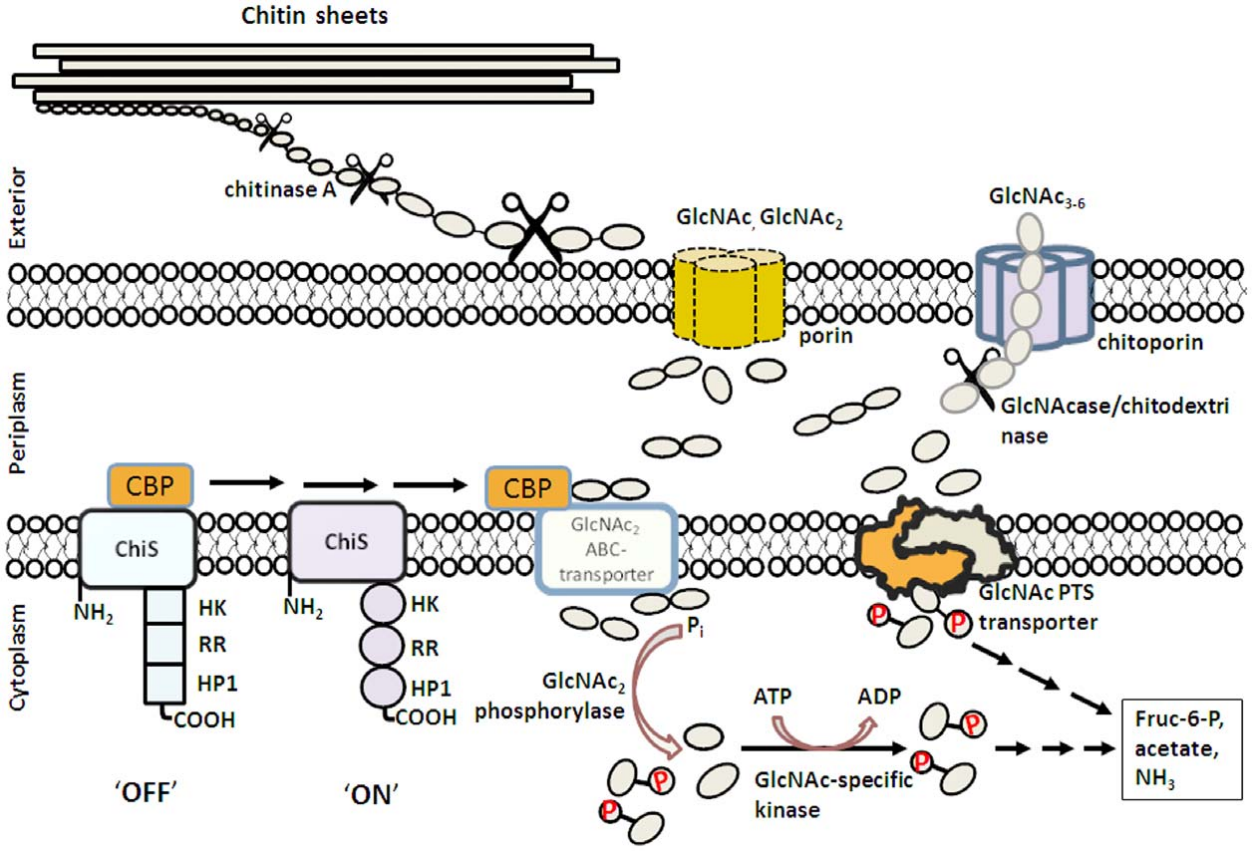
Model of the chitin degradation cascade of the marine bacterium *Vibrio harveyi.* The model was reconstructed from the chitinolytic cascade proposed by Li and Roseman [6]. After chitin degradation by chitinase, the chitin fragments are transported through the outer membrane by diffusion through porin or chitoporin, depending on their sizes. Further enzymatic degradation takes place in the periplasm, producing GlcNAc and GlcNAc_2_. Binding of GlcNAc_2_ to CBD activates the ChiS sensor, producing transcription of the genes under control of the GlcNAc_2_ catabolic operon. GlcNAc is translocated to the cytoplasm by the GlcNAc PTS system, while GlcNAc_2_ is transported through the inner membrane by the GlcNAc_2_ ABC permease. Both products are phosphorylated, and finally converted to Fructose-6-P, acetate and NH_3_.

The chitin catabolic cascade of *Vibrios* is a complex system that involves a cluster of genes in the chitin-induced GlcNAc_2_ operon, which is stringently controlled by a two-component chitin sensor/histidine kinase (also referred to as ChiS sensor) [6], [13]. Fig. 9 summarizes the multiple-step process in the chitin degradation pathway, which involves: i) Chitin binding. Traces of chitooligosaccharides in the surrounding microenvironment are suggested to act as a chemoattractant that triggers adhesion the bacteria to the surface of chitin-containing particles [3], [40]. ii) Chitin degradation. Secretion of chitinases leads to partial degradation of chitin to chitoligosaccharides on the extracellular side of the bacterial cell wall. Endochitinases (mainly chitinase A) were shown to mostly be responsible for chitin degradation [5], [41]. iii) Molecular uptake of chitooligosaccharides. Chitin degradation products presumably permeate the outer membrane of the bacteria through a substrate-specific porin (referred to as “chitoporin or ChiP” [6], [9]. iv) Further breakdown of chitooligosaccharides. In the periplasm, *β*-*N*-acetylglucosaminidase [42] and chitodextrinase [43] degrade the translocated chitin fragments to GlcNAc and GlcNAc_2_. GlcNAc_2_ generated in the periplasm is crucial as it binds to the chitin binding protein (CBP) that is usually attached to ChiS at the outer part of the inner membrane. Dissociation of CBP upon binding to GlcNAc_2_ successively activates the ChiS sensor, which in turn up-regulates expression of the genes that comprise the *chiS* regulon [6], [13]. v) Active transport of GlcNAc and GlcNAc_2_ into cytoplasm. GlcNAc_2_ is transported through the inner membrane by the GlcNAc_2_ ABC-type permease [44], whereas GlcNAc is transported by the phosphoenolpyruvate transferase system (PTS) [45], [46]. vi) Generation of metabolic intermediates. Upon arrival in the cytoplasm, individual dimers are phosphorylated by the cytoplasmic GlcNAc_2_ phosphorylase as follows: GlcNAc_2_ + P_i_ = GlcNAc-6-P + GlcNAc [7]. GlcNAc from the ABC transport system is phosphorylated by a specific GlcNAc kinase [47]. Alternatively, PTS converts GlcNAc to GlcNAc-6-P, concurrently with translocation. As a result, the final forms of intracellular intermediates are fructose-6-P, acetate and NH_3_, which can readily be metabolized as carbon and nitrogen sources for the cells.

Although the chitin degradation pathway of *Vibrios* has been generally accepted [3]–[7], some key issues remain to be clarified concerning the functionality of the proteins involved in the pathway, of which chitoporin is an example. Chitoporin was first identified in *V. furnissii* in 2000 [12]. However, its distinctive function as a chitooligosaccharide-specific channel has not been demonstrated hitherto. To understand the specificity of oligosaccharide transport through the outer membrane, it is necessary to establish the physiological role of this protein. In this study, *V. harveyi* was selected as the source organism for two reasons. First, the mechanisms underlying chitin degradation by chitinase A and *N*-acetylglucosaminidases from *V. harveyi* have been studied in detail by our group [48]–[52]. Second, *V. harveyi* is a fast- growing bioluminescent bacterium through its adaptive ability to grow under anaerobic and respiratory conditions. Therefore, *V. harveyi* contributes significantly to a rapid turnover of chitin in marine ecosystems. It sometimes causes a fascinating phenomenon called ‘milky seas’, in which, during the night, a uniform blue glow is emitted from the seawater. Some glows cover nearly 6,000 square miles of deep oceans. *Ipso facto*, the chitin utilization machinery of *V. harveyi* is expected to work efficiently.

*Vh*ChiP was successfully cloned and the recombinant gene expressed in the *E. coli* system, as verified by mass-spectrometry (Fig. S1) and immunoblotting (Fig. 4A–E). Detection of endogenous expression of chitoporin when the *V. harveyi* cells were grown on chitin-containing medium suggests that the *chiP* gene is regulated by the same control system (the *chiS* regulon, refs 6,13) as the *chi A* gene. We demonstrated previously that in *V. harveyi* expression of the *Chi A* gene was strongly induced by chitin [41].

Single channel recordings revealed that *Vh*ChiP would insert readily into the artificial membranes and behaved as a pore-forming component with a characteristic trimeric closure when high external membrane potentials were applied (Fig. 5). Its structural homology with other porins (Fig. 2) strongly suggests that *Vh*ChiP has 16 β-stranded transmembrane domains, 8 extracellular loops and 8 periplasmic turns, as is observed for most bacterial porins [38]–[39], [53].

BLM current measurements with high time-resolution were used to demonstrate the interaction of chitooligosaccharides with *Vh*ChiP. These are interpreted as indicating oligosaccharide translocation, confirming the specific function of *Vh*ChiP as a chitooligosaccharide-specific porin. The channel was found to interact with the chitosugars to various extents, depending on the sizes and the types of the sugars (Fig. 6). The observation of no fluctuation of ion current on the addition of chitobiose can be explained by the fact that this disaccharide could not permeate through the *Vh*ChiP channel; it may require a general diffusion porin as already described earlier (see Fig. 9). Alternatively, it may permeate so fast that the residence time is too small to lead to well-resolved blocking events. In contrast, the *Vh*ChiP channel was much more sensitive to higher-MW chitosugars (GlcNAc_3–6_). The channel blocking behavior (Figs. 6 and 7) is comparable to the blockage of maltoporin by maltooligosaccharides [19] and also reflects a common characteristic of substrate-specific channels, in which higher-MW oligosaccharides are preferred substrates [32], [54].

BLM measurements revealed no response of *Vh*ChiP to maltopentaose and maltohexaose even at a concentration five-fold greater than that of the chitosugars (Fig. 6). The results of liposome swelling assays additionally confirmed insignificant permeation of other sugars, including raffinose, maltose and sucrose (Fig. 9). These data indicate the high selectivity of the ChiP porin towards chitooligosaccharides. The low sequence identity of between *Vh*ChiP and other sugar-specific porins (less than 20%) (Fig. 1) also demonstrates no detailed structural similarity. *Vh*ChiP appears to be exceptionally specific for chitohexaose, as ion current fluctuation, representing the blockage of individual subunits, was detected at sugar concentrations as low as 125 nM (not shown). The sugar-channel interaction was even stronger at higher concentrations, almost fully blocking all three subunits. Taken together, the results suggest that *Vh*ChiP is a chitooligosaccharide-specific porin. Detailed characterization of channel specificity and binding kinetics towards different chitooligosacchardies has been the subject of our ongoing investigations.

In summary, we employed biochemical assays, together with high-time resolution single channel recordings to address, for the first time, the pore-forming property of chitoporin from the representative *V. harveyi*. The isolated ChiP was found to be highly specific for chitooligosaccharides. The data obtained from this study, therefore, establish the fundamental role of chitoporin in the chitin degradation pathway as the molecular gateway that the marine *Vibrios* employ to efficiently uptake chitooligosaccharides into the cellular interior in order to utilize them as a sole source of energy.

### Accession number

The nucleotide sequence of *V. harveyi* chitoporin has been deposited in the EMBL Nucleotide Sequence Database under accession number HF558985.

## Supporting Information

Figure S1 **Identification of** ***V. harveyi*** **chitoporin by mass spectrometry.** Tryptic peptides were prepared from the outer membrane fraction extracted with 2% (w/v) SDS, followed by 5% (v/v) octyl-POE by in-gel digestion method. The peptides were resolved by nano-LC/MS. The resultant monoisotopic masses were subjected to Mascot search using the NCBINr database for protein identification. Sequences underlined (P1–P9) are identical to nine internal peptides in the translated sequence of *V. harveyi* chitoporin identified in this study.
(TIF)

Figure S2 **Chemical structure of chitin oligosaccharides and maltooligosaccharides.**
(TIF)
